# Husband—Wife, Spinster, Lady

**Published:** 1879-09

**Authors:** 


THE DUCK.—How many does it take to put a duck before two ducks, and a duck between two ducks, and a duck behind two ducks? As any one can see it requires more or less it is not worth while to waste words on the subject.

HUSBAND is the househerd—one who keeps the family together by supporting it. Sometimes the wife is, practically, the husband.

WIFE means a weaver—one who makes the cloth which dresses the family.

SPINSTER is she who spins the yarn which makes the cloth which covers the family.

LADY is compounded of words which mean to give away, to dispense charity to the poor.

